# Foreword: Festschrift in honor of David Dinges, scientist and mentor extraordinaire

**DOI:** 10.1093/sleepadvances/zpad020

**Published:** 2023-04-14

**Authors:** Hans P A Van Dongen, Mathias Basner, Janet M Mullington, Michele Carlin

**Affiliations:** Sleep and Performance Research Center and Department of Translational Medicine and Physiology, Washington State University Health Sciences, Spokane, WA, USA; Unit for Experimental Psychiatry, Division of Sleep and Chronobiology, Department of Psychiatry, University of Pennsylvania Perelman School of Medicine, Philadelphia, PA, , USA; Department of Neurology, Beth Israel Deaconess Medical Center and Harvard Medical School, Boston, MA, USA; Unit for Experimental Psychiatry, Division of Sleep and Chronobiology, Department of Psychiatry, University of Pennsylvania Perelman School of Medicine, Philadelphia, PA, , USA


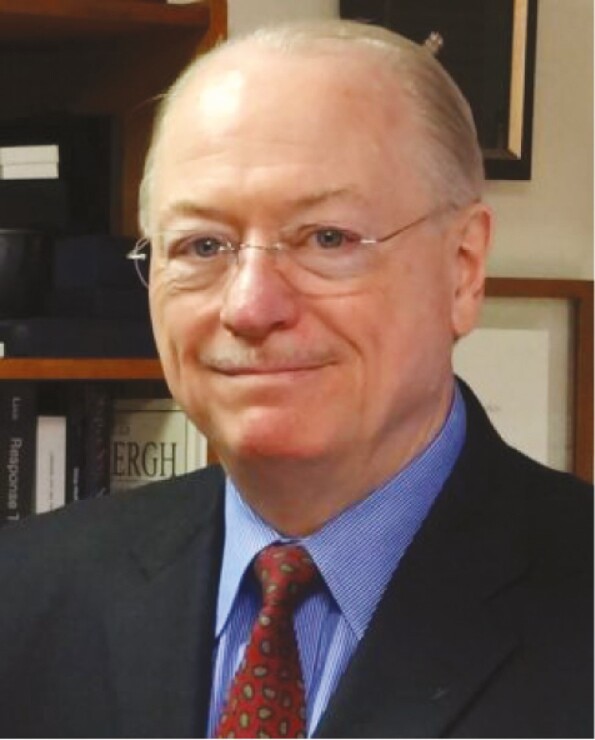


David Dinges, MSc, MA, PhD, is internationally renowned for his extensive studies of the effects of sleep loss and work hours on the neurobehavioral and physiological regulation of human performance and health, with a particular emphasis on demanding, stressful, safety-sensitive settings, such as health care, first responders, transportation, and spaceflight. David’s transition to Emeritus Professor status at the University of Pennsylvania in 2021, celebrated at the height of the COVID-19 pandemic by means of a Zoom videoconference (see Video 1), underscores an illustrious career as a leading sleep and circadian scientist and a cherished mentor to many in the field. With this virtual collection of the journal *SLEEP Advances*, the journal’s editor-in-chief, the Sleep Research Society, and the authors of this Foreword join forces to celebrate David’s many milestone contributions to science and to the scientific community—through the time-tested tradition of a Festschrift, an edited volume honoring a respected academic, presented during their lifetime, containing contributions from colleagues and former trainees [1].

David earned his PhD in Experimental Physiological Psychology from Saint Louis University and, after a brief engagement at George Washington University, joined the Department of Psychiatry at the University of Pennsylvania, where he was tenured in 1993 and promoted to full professor in 1998. He has served as principal investigator of many laboratory and field studies, which were supported by more than 60 federal grants from the National Aeronautics and Space Administration, the National Institutes of Health, the US Departments of Defense and Transportation, as well as grants from foundations and industry. His primary scientific focus has been on identifying how sleep need and its interaction with circadian biology affect human neurobehavioral functions, cognitive performance, operational safety, emotional states, stress responses, endocrine, immune, inflammatory, and metabolic physiology, as well as health.

While it is difficult to choose from David’s extensive publication record (more than 500 papers and chapters to date) for which of his many discoveries, inventions, and ideas to highlight here, probably the most impactful have resulted from his large scale, carefully conducted laboratory studies exposing the cumulative effects of sleep loss on neurobehavioral performance [2–4]. An elegant dose-response study demonstrating the steady build-up of neurobehavioral impairment across consecutive days of sustained sleep restriction [3] stands among the most cited publications in sleep science, reflecting its broad impact on basic and translational sleep science and sleep medicine. Since its publication 20 years ago, this work has had important implications for basic research, raising profound questions about sleep need [5], the temporal dynamics of neurobehavioral functioning [6], and what aspect(s) of sleep provide recuperation [7]. In this Festschrift, David’s former trainee and colleague Siobhan Banks and one of her own trainees summarize the current literature on recovery sleep and suggest next steps for research in this field [8]. Former trainee Janet Mullington, with one of her own trainees and other colleagues, adds a new dimension to this work by examining the influence of sleep timing, with a particular focus on mood [9].

David’s research on sustained sleep restriction also has far-reaching implications for real-world (e.g. operational and clinical) settings, revealing that work schedules that impinge on time for sleep put people at risk of performance impairment and associated errors and accidents [10–12]. This issue is especially pressing in operational settings with little tolerance for error, such as spaceflight—an area in which the contributions of David’s group [13–16] have been particularly influential. For his outstanding contributions to research on astronaut behavioral health with a focus on sleep, circadian rhythms, and neurobehavioral performance, David received NASA’s Distinguished Public Service Medal, which is NASA’s highest honor awarded to a non-government employee, in 2007, and the National Space Biomedical Research Institute’s Pioneer Award in 2016.^1^ In this Festschrift, David’s former trainee and collaborator Terri Weaver and her colleagues review the knowledge base on sleep loss and operational performance as it pertains to astronauts, pilots, and commercial truck drivers [17]. Additionally, the group of Torbjörn Åkerstedt shows that sleep efficiency, but not total sleep time, is a predictor of next-day subjective sleepiness in a population-based cohort of women [18].

With colleagues at the University of Pennsylvania and elsewhere, David has pursued a line of research focused on inter-individual differences in vulnerability to sleep loss. After demonstrating that these inter-individual differences constitute a trait [19], a fruitful search for genetic predictors and other biomarkers ensued [20–22]. Furthermore, David has focused on technological solutions to measure and mitigate the adverse impact of sleep loss on individuals [23–26]. He has played an important role in the development of one of the most sensitive tools available to unobtrusively measure neurobehavioral impairment due to sleep loss: PERCLOS (i.e. percent time of slow eyelid closures) [27]. In this Festschrift, David’s former trainee Takashi Abe provides a review of the present state of evidence on PERCLOS [28].

To study the risks posed by sleep loss with precision, David invented the Psychomotor Vigilance Test (PVT)—a brief (10-min), portable, highly sensitive measure of human behavioral alertness [29, 30], which requires no learning [31]. What exactly makes the PVT so exquisitely sensitive to the effects of sleep loss and circadian rhythmicity has been much investigated and debated [32–37]. David’s influential state instability hypothesis posits that the PVT captures variability in vigilant attention due to the effect of sleep-initiating mechanisms on a person’s endogenous capacity to maintain attention and alertness [32]. Here, David’s former trainee and colleague Hans Van Dongen and the latter’s own former trainee and colleague Kimberly Honn show that while bottom-up variability in vigilant attention is a key contributor [38], top-down attentional control mechanisms also appear to play a role [39].

Colleagues at the Walter Reed Army Institute of Research find that the PVT compares favorably to other tools traditionally used in the sleep field to measure the effects of sleep deprivation (i.e. the multiple sleep latency test and the maintenance of wakefulness test) [40]. Also in this Festschrift, David’s former trainee Julian Lim and his colleagues show that metrics derived from baseline PVT performance can predict vulnerability to performance impairment during sustained sleep restriction [41]. Further, David’s former trainee Daniel Mollicone and his colleagues provide evidence of the utility and ecological validity of an abbreviated (3-min) version of the PVT as a fatigue risk management tool in real world, safety-critical operations [42]. And David’s long-standing colleague Mathias Basner describes the development of an even shorter, adaptive version of the PVT for use in operational settings where time available for taking performance measurements is negligible [43].

Keenly aware that humans often incur sleep loss because of competing demands from work time [44] and social pursuits [45], which puts them at risk of errors and accidents [46], David has also explored a multitude of potential countermeasures. In addition to research on the effectiveness of protected time for sleep [47, 48] and rest breaks [49, 50], his work on nap sleep [32, 51–53] and the associated sleep inertia [54–56] is particularly well known. Pharmacological countermeasures such as caffeine and modafinil/armodafinil have been of interest to him as well [57–61]. Moreover, the impressive amounts of data collected in David’s laboratory have provided a solid foundation for to the development of a biomathematical model of fatigue and performance [62]—a predictive tool that is central in modern approaches to fatigue risk management [63, 64]. Through these and other efforts, David has contributed significantly to policies and practices for fatigue risk management and safety [65–70].

While neurobehavioral function in all of its facets has been central to many of David’s laboratory and field studies, he has also investigated the physiological effects of sleep loss and circadian rhythmicity. This includes studies of metabolism [71, 72], hormones [73–75], inflammatory markers [76–78], and functional brain activation [79–81]. With colleagues at the University of Pennsylvania, this research has extended to obstructive sleep apnea [82–84] and other medical disorders.

The prolific research environment of David’s laboratory has been fertile ground for training and mentoring of students, postdoctoral researchers, and junior faculty. Over the years, David has mentored hundreds of undergraduate, postbaccalaureate, and graduate students, as well as dozens of postdocs and faculty. For 26 years, he taught the hugely popular University of Pennsylvania course “Human Chronobiology and Sleep.” A gifted speaker, he has delivered more than 800 invited lectures in academic settings and at scientific meetings. He has also been quite generous with his time for service to the scientific community, including serving as president of the Sleep Research Society and the World Sleep Federation, as chair or member of numerous advisory and review panels, and as editor-in-chief of the journal *SLEEP* with a 10-year tenure.

David Dinges is a major source of inspiration to the countless individuals who have worked with him as colleagues and collaborators, government and industry partners, or trainees and mentees. For a career retrospective, in the form of an interview with him by Mark Rosekind, see Video 2. It is our hope that this Festschrift stimulates further research and helps move forward the areas in which David’s work has yielded—and continues to yield—so many phenomenal discoveries and insights.

## Footnotes

1 Among David Dinges’ many other honors, the following are particularly noteworthy: Laurence R. Young Space Biomedical Research Award for Contributions to Human Performance in Space (National Space Biomedical Research Institute), Raymond F. Longacre Award for Outstanding Accomplishment in the Psychological & Psychiatric Aspects of Aerospace Medicine (Aerospace Medical Association), Decade of Behavior Research Award (American Psychological Association), Distinguished Scientist Award and Mary A. Carskadon Outstanding Educator Award (Sleep Research Society), Nathaniel Kleitman Distinguished Service Award, Senator Mark O. Hatfield Public Policy Award, and William C. Dement Academic Achievement Award (American Academy of Sleep Medicine), Fellow (American Psychological Association, Association for Psychological Science, American Physiological Society, and Academy of Behavioral Medicine Research), elected member (International Academy of Astronautics), and honorary MA degree in Psychology (University of Pennsylvania).

## Notes

This paper is part of the David F. Dinges Festschrift Collection. This collection is sponsored by Pulsar Informatics, Inc., the Department of Psychiatry in the Perelman School of Medicine at the University of Pennsylvania, the Human Sleep and Cognition Laboratory at Washington State University Health Sciences Spokane, and the Department of Neurology at Beth Israel Deaconess Medical Center.
